# Large-scale analyses of CAV1 and CAV2 suggest their expression is higher in post-mortem ALS brain tissue and affects survival

**DOI:** 10.3389/fncel.2023.1112405

**Published:** 2023-03-02

**Authors:** Brett N. Adey, Johnathan Cooper-Knock, Ahmad Al Khleifat, Isabella Fogh, Philip van Damme, Philippe Corcia, Philippe Couratier, Orla Hardiman, Russell McLaughlin, Marc Gotkine, Vivian Drory, Vincenzo Silani, Nicola Ticozzi, Jan H. Veldink, Leonard H. van den Berg, Mamede de Carvalho, Susana Pinto, Jesus S. Mora Pardina, Mónica Povedano Panades, Peter M. Andersen, Markus Weber, Nazli A. Başak, Christopher E. Shaw, Pamela J. Shaw, Karen E. Morrison, John E. Landers, Jonathan D. Glass, Patrick Vourc’h, Richard J. B. Dobson, Gerome Breen, Ammar Al-Chalabi, Ashley R. Jones, Alfredo Iacoangeli

**Affiliations:** ^1^Social Genetic and Developmental Psychiatry Centre, Institute of Psychiatry, Psychology and Neuroscience, King’s College London, London, United Kingdom; ^2^Department of Biostatistics and Health Informatics, Institute of Psychiatry, Psychology and Neuroscience, King’s College London, London, United Kingdom; ^3^Sheffield Institute for Translational Neuroscience (SITraN), University of Sheffield, Sheffield, United Kingdom; ^4^Department of Basic and Clinical Neuroscience, Maurice Wohl Clinical Neuroscience Institute, Institute of Psychiatry, Psychology and Neuroscience, King’s College London, London, United Kingdom; ^5^Department of Neurosciences, KU Leuven-University of Leuven, Experimental Neurology and Leuven Brain Institute (LBI), Leuven, Belgium; ^6^VIB, Center for Brain and Disease Research, Leuven, Belgium; ^7^Department of Neurology, University Hospitals Leuven, Leuven, Belgium; ^8^UMR 1253, Université de Tours, Inserm, Tours, France; ^9^Centre de référence sur la SLA, CHU de Tours, Tours, France; ^10^Centre de référence sur la SLA, CHRU de Limoges, Limoges, France; ^11^UMR 1094, Université de Limoges, Inserm, Limoges, France; ^12^Academic Unit of Neurology, Trinity Biomedical Sciences Institute, Trinity College Dublin, Dublin, Ireland; ^13^Complex Trait Genomics Laboratory, Smurfit Institute of Genetics, Trinity College Dublin, Dublin, Ireland; ^14^Faculty of Medicine, Hebrew University of Jerusalem, Jerusalem, Israel; ^15^Agnes Ginges Center for Human Neurogenetics, Department of Neurology, Hadassah Medical Center, Jerusalem, Israel; ^16^Department of Neurology, Tel-Aviv Sourasky Medical Centre, Tel-Aviv, Israel; ^17^Sackler Faculty of Medicine, Tel-Aviv University, Tel-Aviv, Israel; ^18^Department of Neurology and Laboratory of Neuroscience, Istituto Auxologico Italiano, IRCCS, Milan, Italy; ^19^Department of Pathophysiology and Transplantation, “Dino Ferrari” Center, Università degli Studi di Milano, Milan, Italy; ^20^Department of Neurology, UMC Utrecht Brain Center, University Medical Center Utrecht, Utrecht, Netherlands; ^21^Instituto de Fisiologia, Instituto de Medicina Molecular João Lobo Antunes, Faculdade de Medicina, Universidade de Lisboa, Lisbon, Portugal; ^22^ALS Unit, Hospital San Rafael, Madrid, Spain; ^23^Functional Unit of Amyotrophic Lateral Sclerosis (UFELA), Service of Neurology, Bellvitge University Hospital, L’Hospitalet de Llobregat, Barcelona, Spain; ^24^Department of Clinical Science, Umeå University, Umeå, Sweden; ^25^Neuromuscular Diseases Unit/ALS Clinic, St. Gallen, Switzerland; ^26^Koc University School of Medicine, Translational Medicine Research Center, NDAL, Istanbul, Turkey; ^27^School of Medicine, Dentistry and Biomedical Sciences, Queen’s University Belfast, Belfast, United Kingdom; ^28^Department of Neurology, University of Massachusetts Medical School, Worcester, MA, United States; ^29^Department of Neurology, Emory University School of Medicine, Atlanta, GA, United States; ^30^Service de Biochimie et Biologie molécularie, CHU de Tours, Tours, France; ^31^National Institute for Health Research Biomedical Research Centre and Dementia Unit at South London and Maudsley NHS Foundation Trust and King’s College London, London, United Kingdom; ^32^Institute of Health Informatics, University College London, London, United Kingdom; ^33^NIHR Biomedical Research Centre at University College London Hospitals, NHS Foundation Trust, London, United Kingdom; ^34^King’s College Hospital, London, United Kingdom

**Keywords:** ALS (Amyotrophic lateral sclerosis), neurodegeneration, differential expression analysis (DEA), survival analysis, caveolin, Cav, CAV1 and CAV2, enhancer variant

## Abstract

**Introduction:** Caveolin-1 and Caveolin-2 (CAV1 and CAV2) are proteins associated with intercellular neurotrophic signalling. There is converging evidence that CAV1 and CAV2 (CAV1/2) genes have a role in amyotrophic lateral sclerosis (ALS). Disease-associated variants have been identified within CAV1/2 enhancers, which reduce gene expression and lead to disruption of membrane lipid rafts.

**Methods:** Using large ALS whole-genome sequencing and post-mortem RNA sequencing datasets (5,987 and 365 tissue samples, respectively), and iPSC-derived motor neurons from 55 individuals, we investigated the role of CAV1/2 expression and enhancer variants in the ALS phenotype.

**Results:** We report a differential expression analysis between ALS cases and controls for CAV1 and CAV2 genes across various post-mortem brain tissues and three independent datasets. CAV1 and CAV2 expression was consistently higher in ALS patients compared to controls, with significant results across the primary motor cortex, lateral motor cortex, and cerebellum. We also identify increased survival among carriers of CAV1/2 enhancer mutations compared to non-carriers within Project MinE and slower progression as measured by the ALSFRS. Carriers showed a median increase in survival of 345 days.

**Discussion:** These results add to an increasing body of evidence linking CAV1 and CAV2 genes to ALS. We propose that carriers of CAV1/2 enhancer mutations may be conceptualised as an ALS subtype who present a less severe ALS phenotype with a longer survival duration and slower progression. Upregulation of CAV1/2 genes in ALS cases may indicate a causal pathway or a compensatory mechanism. Given prior research supporting the beneficial role of CAV1/2 expression in ALS patients, we consider a compensatory mechanism to better fit the available evidence, although further investigation into the biological pathways associated with CAV1/2 is needed to support this conclusion.

## Introduction

Amyotrophic lateral sclerosis (ALS) is a fatal neurodegenerative disease affecting upper and lower motor neurons. It is characterised by the progressive loss of motor function, leading to muscle weakness, difficulty breathing and swallowing, and paralysis. There is currently no treatment, with a mean life expectancy of 3 years (Al-Chalabi and Hardiman, 2013). ALS is comorbid with fronto-temporal dementia (FTD), with an estimated 50% of ALS patients experiencing impaired executive function (Lomen-Hoerth et al., 2003; Strong et al., 2017). These diseases are often conceptualised as two ends of a disease spectrum with a shared pathogenesis and clinical overlap (Phukan et al., 2007; Conlon et al., 2018).

Individuals who have a first-degree relative with ALS are twice as likely than average to develop ALS (Al-Chalabi et al., 2010), and patients with a family history (familial ALS) make up approximately 5%–10% of cases (Zou et al., 2017). A pathogenic variant for familial patients can be identified in over 50% of cases (Turner et al., 2017). However, most cases have no family history (sporadic ALS), and the majority have no identified genetic aetiology. A recent genome-wide association study (GWAS) estimates the narrow-sense heritability of ALS due to SNPs at 8.5% (Van Rheenen et al., 2016). This represents a minimum heritability value based upon the variation of SNPs included in sequencing arrays. Broad sense heritability estimations for ALS vary between 43% and 53% (Ryan et al., 2019; Trabjerg et al., 2020). Of all currently known pathogenic variants, the most common is a hexanucleotide repeat expansion within the *C9orf72* gene, which accounts for 30%–40% of familial cases and 5%–10% of sporadic cases (Brown and Al-Chalabi, 2015; Braems et al., 2020). Individuals with this mutation display an earlier age of onset and faster disease progression (Iacoangeli et al., 2019). A further 5% of sporadic cases are attributable to mutations in *SOD1*, *FUS*, and *TARDBP* genes (Jones et al., 2021).

Despite these known genetic variants, a large proportion of ALS heritability remains unaccounted for. Most ALS genetic studies focus on the study of rare single nucleotide variants (SNVs) and small insertions and deletions (indels) in the coding regions of the genome, or on common single nucleotide polymorphisms (SNPs). As a consequence, structural and rare variants in non-coding regions of the genome are largely under-investigated and could represent a potential source of the missing heritability (Young, 2019; Cooper-Knock et al., 2020; Theunissen et al., 2020).

Caveolin-1 and Caveolin-2 (CAV1 and CAV2, or CAV1/2) genes code for proteins that are associated with the function of membrane lipid rafts. These are regions of low fluidity within the cellular membrane, which act as anchoring points for intercellular signalling (Igarashi et al., 2020). Converging evidence links CAV1 and CAV2 genes to ALS pathology; CAV1 is associated with neuronal survival and is upregulated during induced ischemia in mice, aiding the uptake of extracellular vesicles and reducing apoptosis (Yue et al., 2019). CAV1 may also play a role in the cognitive decline associated with ALS/FTD (Tang et al., 2021), with overexpression increasing neuroplasticity, pro-growth signalling, learning, and memory in mice (Head et al., 2011; Mandyam et al., 2017). Additional evidence using male *SOD1* mice showed that the promotion of neuron-specific CAV1 expression increases body weight and improves longevity and motor function (Sawada et al., 2019). In a subsequent mouse study, subpial administration of synapsin-promoted CAV1 also increased survival, although saw no changes to body weight or motor function (Ichinomiya et al., 2021). Conversely, increased neurodegeneration and synaptic reduction were observed in CAV1 knock-out mice (Head et al., 2010).

In humans, CAV1 coding regions are enriched for ALS-associated variants and CAV1 and CAV2 enhancer mutations are significantly associated with an increased risk of ALS (Cooper-Knock et al., 2020). An expression analysis revealed that two mutations within CAV1 and CAV2 enhancer regions reduced CAV1/2 expression in patient-derived non-neuronal cells, which was supported by CRISPR-Cas9 editing in neuronal cells (Cooper-Knock et al., 2020). Together, evidence from human and mouse studies indicate that CAV1/2 is neuroprotective, and CAV1/2 mutations are a risk factor for ALS pathology, likely as a consequence of reduced gene expression.

In this study, we aim to investigate whether these mutations lead to differences in disease-related phenotypes, as well as changes in ALS risk, and explore whether CAV1/2 expression plays a role in the disease beyond enhancer mutations. In the first set of analyses, we used an RNA-sequencing pipeline to perform expression analysis of the CAV1 and CAV2 genes. The results supported our hypothesis that CAV1 and CAV2 genes would be differentially expressed between ALS cases and controls, with patients showing increased expression. In the second set of analyses, we investigated differences in survival duration and age of onset between ALS patients with and without CAV1/2 enhancer mutations. Considering the evidence that CAV1/2 enhancer mutations reduce CAV1/2 expression and that CAV1/2 expression is beneficial to ALS phenotypes, we hypothesised a reduced survival duration and earlier age of onset in ALS patients who have CAV1 or CAV2 enhancer mutations. Results were opposite to our expectation, showing increased survival duration among carriers of CAV1/2 enhancer mutations. No difference in age of onset was observed between groups.

To confirm whether differential expression of CAV1/2 occurred in neurons specifically, we ran an RNA-seq expression analysis in iPSC-derived motor neurons (MNs) from ALS patients and neurologically normal controls. Additionally, we examined the presence of a correlation between the expression of CAV1/2 in the iPSC-derived MNs and survival, age of onset, and disease progression as measured by the ALSFRS.

## Methods

### Sequencing and clinical data

#### Datasets for RNA-seq differential expression

RNA-seq datasets for the differential expression analyses were obtained from TargetALS at the New York Genome Centre (NYGC; NCBI GEO ID: GSE116622 and GSE124439), the Florida Mayo Clinic (NCBI GEO ID: GSE67196), and the King’s College London and MRC London Neurodegenerative Diseases Brain Bank (Smith et al., 2015; Iacoangeli et al., 2021; Jones et al., 2021).

Sample collection and data generation were previously described (Jones et al., 2021). Briefly, frozen human *post-mortem* samples were used in all cases, and tissue was taken across multiple brain areas. The KCL MRC Brain Bank samples were taken from the primary motor cortex. The Mayo Clinic samples were obtained from the lateral hemisphere of the cerebellum, Brodmann areas 9 and 44 (prefrontal cortex) and Brodmann area 4 (primary motor cortex). The Target alS (NYGC) samples were obtained from the cerebellum, the lateral and medial motor cortex, and various locations within the frontal cortex.

#### Project MinE

Whole genome sequencing and clinical data of ALS cases from Project MinE (data freeze 2) were used for the survival and age of onset analyses (Zhang et al., 2022). Samples were filtered to remove common variants (MAF > 0.01) in the enhancer regions of CAV1 and CAV2 genes, which are defined in Cooper-Knock et al. (2020). Individuals with missing data for sex, survival, and age of onset for the corresponding analysis, or those that failed quality controls (Project MinE ALS Sequencing Consortium, 2018) were removed. This retained 5,987 cases for analysis, including 44 individuals with at least one CAV1 or CAV2 enhancer mutation (individual variants and their frequencies can be found in Supplementary Material). Data generation and whole-genome sequencing quality controls, including principal component analysis, were previously described (Project MinE ALS Sequencing Consortium, 2018; Van Rheenen et al., 2021; Zhang et al., 2022).

#### Answer ALS

Total RNA-seq gene expression profiling of iPSC-derived MNs and phenotype data were obtained for 55 ALS patients and 15 controls from AnswerALS (Baxi et al., 2022). Gene expression was normalized for gene length and then sequencing depth to produce transcripts per kilobase million (TPM). Age of onset and disease status were available for all individuals and these parameters were used to check for the correlation between the expression of top-ranked RefMap ALS genes and age at disease onset.

### Data analysis

#### RNA-seq differential expression analysis

**Figure 1:**
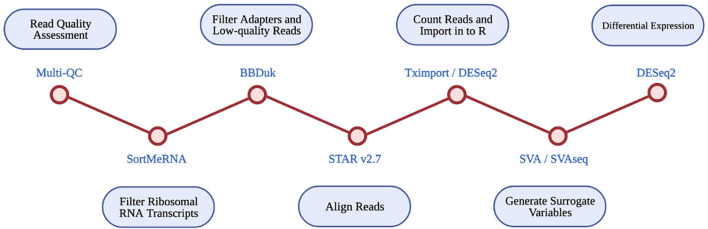
Diagrammatic representation of RNA-seq differential expression pipeline. Each RNA-seq step is shown in the blue circles, with the tool used at each step given beside each red circle.

An RNA-seq based differential expression analysis was performed for CAV1 and CAV2 genes on samples across three datasets. A detailed protocol of library preparation is described by Tam et al. (2019) for TargetALS samples, Prudencio et al. (2015) for Mayo Clinic samples, Prudencio et al. (2015) and Jones et al. (2021) for the KCL MRC Brain Bank samples. Figure 1 illustrates the stages performed in the RNA-seq analysis.

Multi-Q23 was used for all datasets to assess read quality pre- and post- alignment. The removal of ribosomal RNA transcripts was achieved by filtering with SortMeRNA, using rRNA databases. BBDuk was used to filter adapters and low-quality reads. RNA-seq reads were aligned with STAR v2.7 using the GRCh37.89 reference genome.

Read counts were imported into R using Tximport and DESeq2. Only transcripts with at least 10 reads were retained for analysis. Available data for disease status, gender, quintiles of age, quintiles of PMI, RIN, and flow-cell were imported into R. SVA and SVAseq were used to generate surrogate variables for each sample, which estimate expression heterogeneity. These were included as covariates in subsequent analyses to control for unaccounted confounding factors such as cell heterogeneity and extraneous variation.

Raw read counts were supplied to DESeq2, which was used to perform a differential expression analysis across ALS cases and controls. Differential expression was estimated using log_2_ fold-change, a wald test, and FDR *p*-value correction. Analyses were run using covariates of age, gender, *post-mortem* delay, RIN, and surrogate variables, where data was available.

The final differential expression results were meta-analysed for each brain tissue type using the Stouffer method (Stouffer et al., 1949). This uses the p-value, sample size, and log_2_ fold-change from each dataset to produce meta-analysed test statistics, and considers the direction of effect.

#### Project MinE survival and age of onset analyses

Multiple cox proportional hazard survival analyses were run and visualised in R using the *survival* and *survminer* packages. These analyses were to assess whether the presence of CAV1/2 enhancer mutations impacts patient survival. Analyses were run with sex at birth and age of onset as covariates, using individuals with no CAV1/2 mutations together with: CAV1 mutations only, CAV2 mutations only, and individuals with mutations in either gene.

C9-related ALS is characterised by different clinical presentations (Al-Chalabi et al., 2016, 2017), earlier age of onset, and faster disease progression compared to non-C9 ALS, suggesting a separate disease mechanism (Iacoangeli et al., 2019). Analyses were therefore run with and without individuals carrying a pathogenic repeat expansion of the* C9orf72* gene (Iacoangeli et al., 2019) to assess whether increasing sample homogeneity would reveal a stronger effect of CAV1/2 mutations on survival. Analyses were additionally run excluding samples from patients with other well-known ALS mutations (*SOD1, FUS, TARDBP*), and matching samples based on nationality. Finally, survival analyses were run when stratifying samples by type of CAV enhancer mutation (CAV1 or CAV2).

A second set of analyses were run to determine whether CAV1/2 status affected age of onset, using sex at birth as a covariate. These were linear regression and cox proportional hazard models, run in R using the survival package. Analyses were run with and without carriers of a pathogenic* C9orf72* repeat expansion. They compared samples with no CAV1/2 mutation to: (1) samples with CAV1 enhancer mutations; (2) samples with CAV2 enhancer mutations; and (3) samples with either mutation.

## Results

### Samples and datasets

#### Differential expression analysis datasets

**Figure 2:**
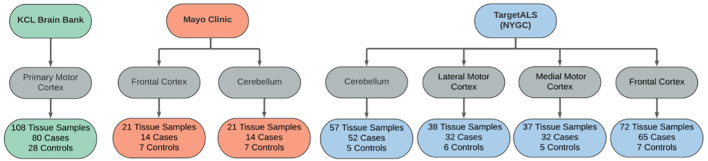
Sample overview across the RNAseq datasets used in the differential expression analyses. Datasets were obtained from the KCL Brain Bank (green), Mayo Clinic (orange), and TargetALS (NYGC; blue).

Samples were matched across disease status by age and sex within each dataset, where data was permitted. Cases were comprised of samples from sporadic and familial ALS patients, including *C9orf72-* and *SOD1*-associated ALS. Control samples were obtained from individuals with non-neurological or non-ALS disease. An outline of each dataset is provided in Figure 2.

#### Project MinE dataset for CAV1/2 enhancer mutation analyses

**Figure 3:**
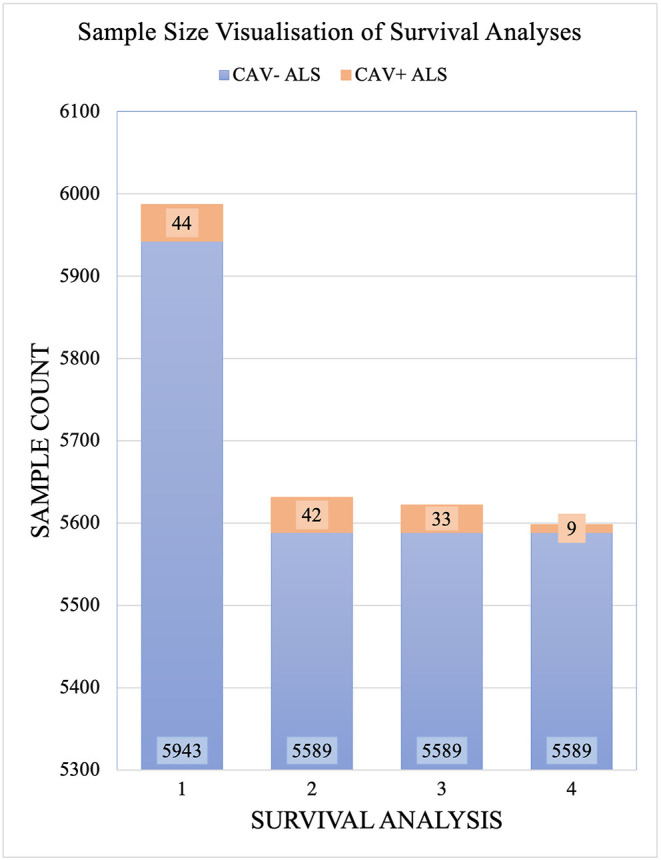
Sample sizes for each Project MinE survival analysis. Samples are divided by those with CAV1/2 enhancer mutations (orange) and without (blue). Analyses are: Full dataset including all rare mutations; excluding C9orf72 samples; and with CAV1 and CAV- samples only with CAV2 and CAV- samples only. CAV- refers to patients who do not carry CAV1/2 enhancer mutations and CAV+ refers to those who do.

CAV1/2 enhancer variants of MAF > 0.01 in gnomAD were removed prior to analysis. 5,987 samples passed the quality controls and were used for analysis. Of these, 356 were carriers of the *C9orf72* repeat expansion. In total, 44 patients had at least one CAV1/2 enhancer mutation, of which, 34 were carriers of CAV1 mutations, and 10 were carriers of CAV2 mutations. Figure 3 shows sample sizes for the four primary Project MinE survival analyses.

### Bulk RNAseq reveals higher expression of CAV1 and CAV2 in ALS patient tissue compared to controls

**Figure 4:**
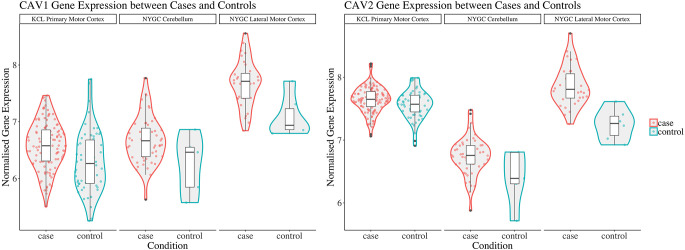
This figure shows violin plots of significant gene expression for CAV1 and CAV2 between cases and controls. The X-axis indicates tissue/dataset combination and case/control status. The Y-axis is normalised gene expression. Coloured dots inside violin plots are jittered gene expressions for each sample. Boxplots inside each violin plot show gene expression for each category. Violin plot colour: Condition (case: red; control: blue). Note that CAV1 differential expression in the lateral motor cortex is significant only to *p* < 0.1. Violin plots for all analyses are available in Supplementary Figure 1.

Considering converging evidence that CAV1/2 genes are neuroprotective and the previous association between ALS disease status and CAV1/2 enhancer regions, we hypothesised that CAV1 and CAV2 genes would be differentially expressed between ALS patients and controls within brain tissue. Results from the differential expression analysis for CAV1 and CAV2 are outlined in Table 1 and shown in violin plots in Figure 4. CAV1 showed statistically significant differential gene expression within the KCL primary motor cortex (Log2FC = 0.396, *p* = 0.04) and the NYGC cerebellum (Log2FC = 0.751, *p* = 0.02). CAV2 was differentially expressed in the primary motor cortex within the KCL BrainBank sample (Log2FC = 0.183, *p* = 0.01), in addition to the cerebellum (Log2FC = 0.669, *p* = 0.004) and lateral motor cortex (Log2FC = 0.691, *p* = 0.029) within Target alS (NYGC) samples. Dataset-tissues almost universally showed a positive log_2_ fold-change (with the exception of the NYGC frontal cortex), suggesting that CAV1/2 is consistently upregulated among ALS cases. This direction of effect is contrary to previous evidence if we conclude that a higher expression level in cases corresponds to gene expression increasing ALS risk. However, this aligns with a compensatory model, in which expression of CAV1/2 genes is increased to mitigate ALS-related pathology.

**Table 1:** The table outlines the differential expression (Log_2_-fold change) for CAV1 (blue) and CAV2 (green) across brain tissues and datasets, *p < 0.05.

Dataset	Tissue	Cases	Controls	CAV1		CAV2	
				Log_2_ Fold-Change	*p*-value	Log_2_ Fold-Change	*p*-value
KCL BrainBank	Primary Motor Cortex	80	28	0.396	0.04*	0.183	0.01*
Mayo Clinic	Frontal Cortex	14	7	0.019	0.937	0.066	0.722
	Cerebellum	14	7	0.134	0.71	0.233	0.431
Target alS (NYGC)	Cerebellum	52	5	0.751	0.022*	0.669	0.004*
	Lateral Motor Cortex	32	6	0.762	0.091	0.691	0.029*
	Medial Motor Cortex	32	5	0.401	0.345	0.054	0.875
	Frontal Cortex	65	7	0.202	0.448	−0.232	0.22

Log_2_ fold-change for CAV1 and CAV2 were in a consistent direction across all datasets and tissues except for the CAV2 NYGC frontal cortex. For this reason, a Stouffer meta-analysis was run for the motor cortex, frontal cortex, and cerebellum, the results of which are shown in Table 2. Two TargetALS NYGC tissue regions were available within the motor cortex, the lateral and medial motor cortex, of which only the lateral motor cortex reached statistical significance (Log2FC = 0.691, *p* = 0.029). These datasets were separately meta-analysed with the KCL Brainbank dataset. These analyses were statistically significant for both CAV1 and CAV2 genes, and all showed a large log2 fold-change over 2.

**Table 2:** Stouffer meta-analysis of differential expression data, *p < 0.05.

Datasets	Tissue	Sample size 1	Sample size 2	CAV1		CAV2	
				Log_2_ Fold-Change	*p*-value	Log_2_ Fold-Change	*p*-value
KCL + NYGC (Lateral)	Motor Cortex	108	38	2.499	0.012*	3.155	0.002*
KCL + NYGC (Medial)	Motor Cortex	108	37	2.249	0.025*	2.488	0.013*
Mayo + NYGC	Frontal Cortex	21	72	0.751	0.453	−1.078	0.281
Mayo + NYGC	Cerebellum	21	57	2.280	0.023*	2.969	0.003*

### CAV1/2 expression is higher in iPSC-derived motor neurons from ALS patients

Bulk RNA-seq in *post-mortem* brain tissue has shown that expression of both *CAV1* and *CAV2* genes is higher in ALS patients compared to controls. Enhanced CAV1 expression has previously been associated with neuroprotection (Sawada et al., 2019) and reduced CAV1 expression has been associated with risk for ALS (Cooper-Knock et al., 2020). Therefore, the observed higher expression of CAV1 and CAV2 might represent a compensatory reaction to neurotoxicity. However, the bulk RNA-seq analysis does not allow us to determine which cell types are responsible for observed changes in *CAV1/2* expression. To address this, we analysed gene expression in iPSC-derived MNs from ALS patients (*n* = 55^1^) and neurologically normal controls (*n* = 15). Mean expression of both genes was higher in ALS patients compared to controls although this difference was not statistically significant (CAV1: mean ALS = 1.46 TPM, mean control = 1.3 TPM, *t* = 0.48, Log2FC = 0.1575, *p* = 0.31. CAV2: mean ALS = 1.67 TPM, mean control = 1.39 TPM, *t* = 1.43, Log2FC = 0.2647, *p* = 0.08).

### Correlation analyses between CAV1/2 expression and phenotypic measures in answer ALS

Using RNAseq from iPSC-derived MN, we examined the association between CAV1/2 expression and phenotypic measures. An outline of these results is shown in Table 3. Age of onset was quantified in days; there was no significant correlation between CAV1/2 expression and age of onset (Pearson correlation *p* > 0.05). Survival was measured in days from the date of onset to death and censored samples were not included because of the lack of longitudinal data; the date of death was available for 27 ALS patients. Cox proportional hazards model was used to determine whether survival was significantly correlated with CAV1/2 expression. The first 10 principal components were used as covariates to control for population structure. Neither CAV1 (*p* = 0.96) nor CAV2 (*p* = 0.70) were significantly associated with survival in this cohort.

**Table 3:** Results of AnswerALS RNA-seq expression and phenotypic correlation analyses for CAV1 (blue) and CAV2 (green).

Analysis	Test	CAV1			CAV2		
		Coefficient	*t*	*p*-value	Coefficient	*t*	*p*-value
iPSC Gene Expression	*t*-test	NA	0.48	0.31	NA	1.43	0.08
Age of Onset	Pearson Correlation	0.13	NA	0.31	−0.19	NA	0.39
Survival	Cox Proportional Hazard	−0.02	NA	0.96	0.70	NA	0.21
Disease Progression (ALSFRS Score)	Pearson’s Correlation	−0.11	−0.72	0.76	−0.27	−1.78	0.04

**Figure 5:**
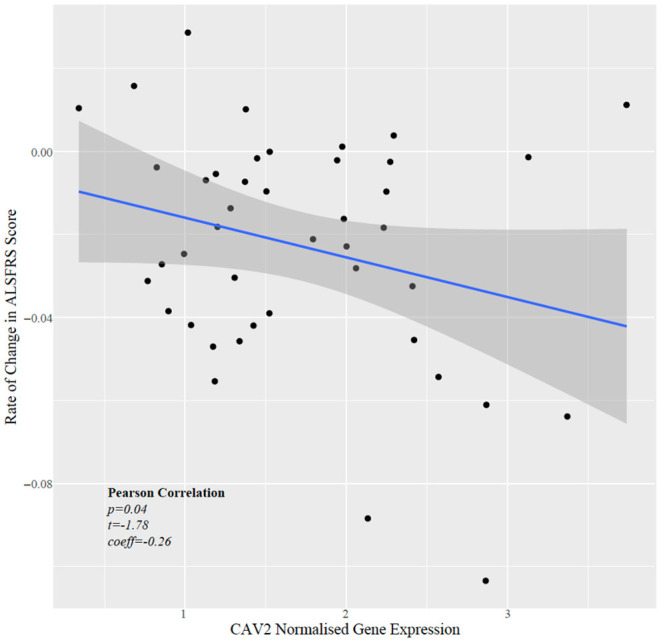
Scatter plot showing normalised CAV2 gene expression against rate of change in the ALSFRS.

Next, we tested whether CAV1/2 expression was correlated with the rate of change in ALSFRS, which is a measure of the rate of disease progression. The ALSFRS was measured longitudinally between 2 and 10 times (with a median of four measurements). The delta-ALSFRS was calculated using linear regression based upon patient visit time and was available for 43 ALS patients. CAV2 expression but not CAV1 expression was negatively correlated with the rate of change of ALSFRS score (Figure 5); iPSC-derived MN with higher CAV2 expression were derived from patients with a faster rate of decline in the ALSFRS (Pearson correlation *p* = 0.04, *t* = −1.78, *r* = −0.27). In view of our previous data, this could suggest that a compensatory increase in CAV2 expression is highest in patients with more rapid disease progression. It is interesting that CAV1 has been previously associated with neuroprotection but was not significant in this test which may indicate opposing forces of compensatory upregulation with more aggressive disease and a therapeutic effect slowing disease progression.

### Survival analyses in project MinE

**Figure 6:**
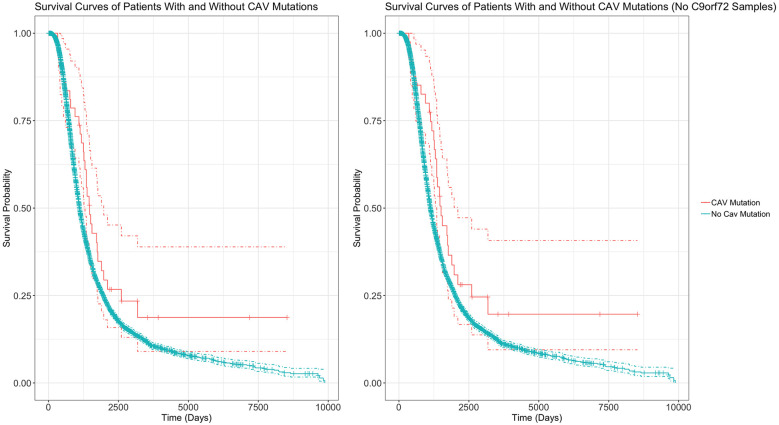
Survival Curves comparing survival of patients with vs without any CAV1/2 mutation. The left graph is based upon data from Analysis 1, inclusive of patients with C9orf72 repeat expansion. The right graph is from Analysis 2, with C9orf72 samples removed. Patients with CAV1/2 mutations have a longer survival time (C9orf72-inclusive analysis: median survival difference of 345 days. See Table 5 for a full descriptive summary). Y-axis is the fraction of surviving sample. X-axis is time in days. Dashed lines indicate 95% confidence intervals. Orthogonal lines indicate death or censoring event. Graphs exclude 22 samples from patients surviving over 10,000 days to improve scaling. Complete graphs are available in Supplementary Figure 2.

Table 4 outlines the results from four of these survival analyses. In the first set of analyses (1–2), we tested the difference in survival of the patients carrying a mutation in the enhancer of either gene (CAV1/2) against non-carriers (Figure 6). The decision was made to combine CAV1 and CAV2 enhancer mutations due to their related biological function, co-expression, overlapping enhancers, and to maximise the statistical power. CAV1/2 mutations were significantly associated with longer survival (HR = 0.694, *p* = 0.043; HR = 0.674, *p* = 0.034). This was the case irrespective of whether *C9orf72* samples were included or removed.

**Table 4:** Breakdown of results across four survival analyses.

	C9orf72	CAV Enhancer Mutations	CAV+ ALS	CAV− ALS		Hazard Ratio (95% CI)	Standard Error	*p*-value
1	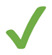	CAV1 and CAV2	44	5,943	**CAV**	0.694 (0.487, 0.988)	0.180	0.043*
					**Age of onset**	1.000	0.000	<0.001^***^
					**Sex at birth**	1.075 (1.013, 1.141)	0.031	0.018*
2	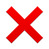	CAV1 and CAV2	42	5,589	**CAV**	0.674 (0.468, 0.971)	0.186	0.034*
					**Age of onset**	1.000	0.000	<0.001^***^
					**Sex at birth**	1.085 (1.020, 1.155)	0.032	0.010**
3	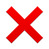	CAV1	33	5,589	**CAV**	0.729 (0.484, 1.099)	0.201	0.131
					**Age of onset**	1.000	0.000	<0.001^***^
					**Sex at birth**	1.084 (1.019, 1.154)	0.032	0.011*
4	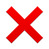	CAV2	9	5,589	**CAV**	0.523 (0.235, 1.164)	0.409	0.112
					**Age of onset**	1.000	0.000	<0.001^***^
					**Sex at birth**	1.088 (1.022, 1.158)	0.032	0.008**

The left side of the table displays the inclusion criteria of each analysis, and the right side displays the results. The first two columns specify whether samples with a C9orf72 mutation have been included (green tick) or excluded (red cross). CAV+ denotes the number of samples with CAV1/2 enhancer mutations, and CAV- indicates the sample size of those without CAV1/2 enhancer mutations. *p < 0.05; **p < 0.01; ^***^p < 0.001.

The following analyses were then stratified by the presence of CAV1 or CAV2 enhancer mutations. These analyses excluded *C9orf72* samples. Although not significant, the effects on survival of CAV1 and CAV2 enhancer mutations were similar and consistent with the analyses 1–2. This supports our initial choice to aggregate them to increase statistical power based on the hypothesis that mutations in the enhancers of both genes have a similar role in ALS. Descriptive statistics for these analyses are available in Table 5.

**Table 5:** This table shows the mean, standard deviation, median, and range of survival in days for individuals with uncensored data.

		**Mean**	**SD**	**Median**	**Range**
1	CAV	1,285.7	667.99	1,303	2,858
	No CAV	1,229.26	1,061.51	958	16,811
	Difference	56.44		345	
2	CAV	1,336.85	657.76	1,351	2,830
	No CAV	1,243.51	1,086.16	964	16,811
	Difference	93.34		387	
3	CAV	1,243.51	1,086.16	964	16,811
	No CAV	1,231.91	601.85	1,303	2,250
	Difference	11.6		−339	
4	CAV	1,243.51	1,086.16	964	16,811
	No CAV	1,739.12	764.35	1,442.5	2,055.25
	Difference	−495.61		−478.5	

Analyses correspond to the rows in Table 4. Analyses 1 and 2 are including and excluding patients with the C9orf72 repeat expansion, respectively. Analysis 3 and 4 are stratified by CAV1 and CAV2 enhancer mutation, and do not include C9orf72 mutations.

### Age of onset in project MinE

Similarly to the survival analyses, each age of onset analysis was performed using differing inclusion criteria. Table 6 displays the results for all age of onset analyses and their inclusion criteria. Cox Proportional hazards model was used for each analysis, setting the event status indicator to 1 (the event has occurred) for each sample. In parallel, a linear regression was performed using the same inclusion criteria as analysis 1. No analysis found any effect of CAV1/2 mutation on the age of onset.

**Table 6:** The left side of the table describes the inclusion criteria and CAV+/CAV− sample size; the right size shows results from age of onset analyses using Cox proportional hazards and linear regression models; ^***^p < 0.001.

	**C9orf72**	**CAV mutations**	**CAV+ ALS**	**CAV− ALS**		**Hazard Ratio (95% CI)**	**Standard Error**	***p*-value**
1	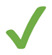	CAV1 and CAV2	44	5,943	**CAV**	1.034 (0.826, 1.296)	0.115	0.768
					**Sex at birth**	0.864 (0.820, 0.910)	0.026	<0.001^***^
2	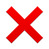	CAV1 and CAV2	42	5,589	**CAV**	1.036 (0.823, 1.303)	0.117	0.764
					**Sex at birth**	0.859 (0.814, 0.906)	0.027	<0.001^***^
3	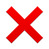	CAV1	33	5,589	**CAV**	1.078 (0.765, 1.518)	0.175	0.669
					**Sex at birth**	0.858 (0.813, 0.905)	0.027	<0.001^***^
4	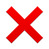	CAV2	9	5,589	**CAV**	0.864 (0.449, 1.662)	0.334	0.662
					**Sex at birth**	0.857 (0.813, 0.905)	0.027	<0.001^***^
Linear Regression						*t*-value	Standard Error	*p*-value
5	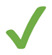	CAV1 and CAV2	44	5,943	**CAV**	−0.425	1.916	0.671^***^
					**Sex at birth**	6.476	0.334	<0.001^***^

## Discussion

We report increased expression of CAV1 and CAV2 in ALS cases when compared to controls using bulk RNA sequencing from *post-mortem* brain tissue samples. Statistically significant differential expression was found in the KCL Brainbank and Target alS (NYGC) samples, but not in Mayo Clinic samples, although the direction of effect was consistent. Non-significant results may be due to a lack of power, as the sample size was substantially smaller in the Mayo Clinic samples than the other datasets. Additionally, meta-analyses revealed significant differences within the cerebellum and motor cortex for both CAV1 and CAV2 expression, but not the frontal cortex. One possible interpretation is that overexpression of CAV1/2 genes increases ALS risk. However, this is inconsistent with evidence that CAV1/2 expression is protective in ALS (Head et al., 2011; Cooper-Knock et al., 2020) and more generally promotes neuronal growth and improves motor function (Egawa et al., 2017, 2018). An alternative interpretation consistent with previous literature is that the gene upregulation is indicative of a compensatory mechanism; CAV1/2 expression is increased as a response to ALS pathology, which affords greater protection.

Survival analyses showed that among ALS patients, carriers of CAV1/2 enhancer mutations had longer survival compared to non-carriers, with a median survival difference of 345 days in the Project MinE dataset. No correlation was demonstrated between gene expression and survival in the AnswerALS iPSC-derived MNs, although this analysis was limited by the small sample size. We observed a negative correlation between CAV2 expression and the rate of change in the ALSFRS in the iPSC-derived MNs. Given the seemingly protective role of CAV1/2, it was expected that mutations in CAV1/2 enhancers, which purportedly decrease CAV1/2 expression, would in turn reduce survival. We consider two possible explanations for observing the opposite outcome. CAV1/2 enhancer mutations exist in non-coding regions and have an unknown impact on gene expression. Cooper-Knock and colleagues (Cooper-Knock et al., 2020) ran an expression analysis using a single CAV1/2 enhancer mutation (chr7:116222625:T > C), finding an association with reduced CAV1/2 expression in patient-derived neuronal cells. However, this is not sufficient evidence to conclude the global effect of CAV1/2 mutations on expression, as enhancer mutations may also increase gene expression (Corradin and Scacheri, 2014; Sur and Taipale, 2016). The effects of other variants on gene expression may account for the increased survival duration that we observed. Further investigation into the of CAV1/2 enhancer mutations on gene expression would be beneficial to build evidence for or against this interpretation.

An alternative hypothesis is that patients with CAV1/2 mutations represent a subset of ALS patients with a less aggressive phenotype. In this framework, CAV1/2 enhancer mutations reduce CAV1/2 expression, leading to dysfunctional neuronal signalling and accelerated neurodegeneration. However, the dysfunction associated with CAV1/2 is on average less severe than non-CAV-related ALS phenotypes, leading to the longer survival time found in our analyses. It is more likely that rare variants occurring within enhancer regions are deleterious, leading to reduced function of the enhancer and therefore reduced expression than to improve function and increase CAV1/2 expression. This prior expectation makes this interpretation more biologically plausible.

Whether or not CAV1/2 enhancer mutations increase or decrease CAV1/2 gene expression, both align with the “compensatory model” of CAV1/2 overexpression in ALS patients. If CAV1/2 are neuroprotective and are upregulated to compensate for ALS pathology, CAV1/2 enhancer mutations which increase expression simply boost this effect, leading to increased survival. If these mutations decrease expression and subsequently increase neurodegeneration, the “increased survival” we observe among patients with CAV1/2 enhancer mutations may be explained by CAV-mediated ALS being on average less severe than non-CAV ALS.

Individuals with CAV1/2 mutations represent a small but relevant proportion of ALS patients (0.7%). Our results add to an increasing body of evidence linking CAV1 and CAV2 genes to ALS, help to elucidate the role of their enhancer mutations and gene expression in ALS, and support the positioning of CAV1/2 genes as potential targets for the development of treatment. However, further research into the functional effect of CAV1/2 mutations is needed to clarify their role in the pathogenesis of ALS.

## Data availability statement

Publicly available datasets were analyzed in this study. This data can be found here: GSE116622: https://www.ncbi.nlm.nih.gov/geo/query/acc.cgi?acc=GSE116622 GSE124439: https://www.ncbi.nlm.nih.gov/geo/query/acc.cgi?acc=GSE124439 GSE67196: https://www.ncbi.nlm.nih.gov/geo/query/acc.cgi?acc=GSE67196 Project MinE: https://www.projectmine.com/research/data-sharing/ AnswerALS: https://dataportal.answerals.org/data-search.

## Ethics statement

The studies involving human participants were reviewed and approved by the Institute of Psychiatry, Psychology & Neuroscience, King’s College London, and the MRC London Neurodegenerative Diseases Brain Bank, in addition to the following ethical committees: The Netherlands: University Medical Center Utrecht Medical Ethics Committee, Utrecht, Netherlands. UK MNDA Biobank: Trent University Medical Ethics Committee. UK (Sheffield): Yorkshire and the Humber - Sheffield Research Ethics Committee. Turkey: Ethics Committee on Research with Human Participants (INAREK) at Bogazici University, Istanbul, Turkey. Belgium: Ethical Committee of University Hospital Leuven. Ireland: Beaumont Hospital Research & Ethics Committee. Spain (Madrid): Comité de ética de la Investigación del Hospital Carlos III. Spain (Barcelona): Bellvitge University Hospital Ethics Committee, Barcelona, Spain. United States: Committee for the Protection of Human Subjects in Research of the University of Massachusetts Medical School, Worcester, USA. France (Paris): Medical Research Ethics Committee of “Assistance Publique-Hôpitaux de Paris”. France (Tours): The ethics committee of Tours Hospital, France. France (Limoges): Ethics committee of Limoges University Hospital, France. Sweden: Regional Ethical Review Board in Umeå. Israel (Tel-aviv): The Institutional Review Board of Tel Aviv Sourasky Medical Center, Israel. Israel (Jerusalem): Hadassah University Hospital IRB board. Portugal: The Local Research Ethics Committee at the Faculty of Medicine, University of Lisbon, Lisbon, Portugal. Italy: Ethical Committee of Città della Salute Hospital, Torino, Italy. Switzerland: Kantonale Ethikkomission des Kantons St. Gallen, Switzerland. Australia: Sydney South West Area Health Service Human Research Ethics Committee; HREC at the different sites: University of Sydney, Western Sydney Local Health District, Royal Brisbane and Women Hospital Metro North, South Metropolitan Health Service, Macquarie University, QIMR Berghofer Medical Research Institute, University of New South Wales and the University of Melbourne. The patients/participants provided their written informed consent to participate in this study.

## Author contributions

BA, JC-K, AJ, and AI contributed to concept, design of the study, running the analyses, and drafted the manuscript. All authors contributed to the article and approved the submitted version.

## Footnotes

^1^ https://www.answerals.org/

## Supplementary material

Supplementary Figure 1 Violin plots of gene expression for CAV1 and CAV2 between cases and controls. The X-axis indicates tissue/dataset combination and case/control status. The Y-axis is normalised gene expression. Coloured dots inside violin plots are jittered gene expressions for each sample. Boxplots inside each violin plot show gene expression for each category. Violin plot colour: Condition (case: red; control: blue).

Supplementary Figure 2 Uncropped survival curves comparing survival of patients with vs without any CAV1/2 mutation. The top graph is based upon data from Analysis 1, inclusive of patients with C9orf72 repeat expansion. The bottom graph is from Analysis 2, with C9orf72 samples removed. Patients with CAV1/2 mutations have a longer survival time (C9orf72-inclusive analysis: median survival difference of 345 days. See Table 5 for a full descriptive summary). Y-axis is the fraction of surviving sample. X-axis is time in days. Dashed lines indicate 95% confidence intervals. Orthogonal lines indicate death or censoring event.

Supplementary Table 1 List of CAV1/2 enhancer variants and their frequency within the Project MinE dataset.
